# Direct and Reverse Pluronic Micelles: Design and Characterization of Promising Drug Delivery Nanosystems

**DOI:** 10.3390/pharmaceutics14122628

**Published:** 2022-11-28

**Authors:** Almudena Naharros-Molinero, María Ángela Caballo-González, Francisco Javier de la Mata, Sandra García-Gallego

**Affiliations:** 1 Berlimed S.A., Francisco Alonso, 7, 28806 Alcalá de Henares, Spain; 2 Department of Organic and Inorganic Chemistry, Faculty of Sciences, and Research Institute in Chemistry “Andrés M. del Río” (IQAR), Universidad de Alcalá, 28801 Alcalá de Henares, Spain; 3 Institute Ramón y Cajal for Health Research (IRYCIS), 28034 Madrid, Spain; 4 Networking Research Center on Bioengineering, Biomaterials and Nanomedicine (CIBER-BBN), 28029 Madrid, Spain

**Keywords:** Pluronic, poloxamer, direct micelle, reverse micelle, drug delivery

## Abstract

Pluronics are a family of amphiphilic block copolymers broadly explored in the pharmaceutical field. Under certain conditions, Pluronics self-assemble in different structures including nanosized direct and reverse micelles. This review provides an overview about the main parameters affecting the micellization process of Pluronics, such as polymer length, fragments distribution within the chain, solvents, additives and loading of cargo. Furthermore, it offers a guide about the most common techniques used to characterize the structure and properties of the micelles. Finally, it presents up-to-date approaches to improve the stability and drug loading of Pluronic micelles. Special attention is paid to reverse Pluronics and reverse micelles, currently underexplored in the literature. Pluronic micelles present a bright future as drug delivery agents. A smart design and thorough characterization will improve the transfer to clinical applications.

## 1. Introduction

In the field of drug delivery, nanomaterials have demonstrated great potential and are entering the era of personalized medicine [1]. Lipidic, polymeric and inorganic nanoparticles (NPs) are among the most explored drug delivery systems. They offer important technological advantages including high loading capacity, prolonged stability of the drug and tunable design to address different routes of administration. 

Pluronics^®^ are a family of amphiphilic block copolymers, broadly used in pharmaceutical and clinical applications due to their low immunogenicity and exceptional biocompatibility [2]. Certain Pluronics have been approved by the U.S. Food and Drug Administration (FDA) and the European Medicines Agency (EMA) for their use as surfactants, solubilizers, emulsifying agents and absorption enhancers [2]. In addition, they are employed as matrices and carriers, according to their most characteristic properties: temperature-dependent gelation and micellization. These properties enable the transport of active pharmaceutical ingredients (APIs), especially those with poor water solubility.

Under certain conditions, Pluronics self-assemble into different supramolecular structures, where nanosized micelles stand out. Micelles are defined as colloidal NPs with a core–shell architecture and sizes in the range of 10–100 nm [3]. The amphiphilic unimers of Pluronic polymers self-assemble at critical micelle concentrations (CMC) and form either direct micelles (DMs) or reverse micelles (RMs) depending on the external solvent. In general, polymeric micelles are outstanding drug delivery vehicles with three unmatched advantages [4,5]: Firstly, their relatively small size, which favors blood circulation, tissue penetration, cell uptake and in vivo performance of encapsulated drugs, as the preferred size range for drug delivery is 40–50 nm [6]. Secondly, the polymer structure regulates the drug release preventing a rapid clearance, prolonging its circulation time and decreasing the toxicity of the loaded drug. Thirdly is the feasibility of large-scale manufacturing, as it is the simplest assembled entity with a well-defined molecular structure. These advantages promoted the global acceptance of micelles as a first-line drug formulation technology. 

Beyond the capacity of Pluronics to form micellar nanocarriers, these copolymers have been used in the modification of a myriad of other NPs, generating hybrid structures with mixed properties: polymeric NPs, metal NPs, nano-suspensions, liposomes, dendrimers, nanogels, polymeric micelles and solid lipid NPs [7]. Furthermore, they have been functionalized with different moieties such as pH-sensitive, biological-responsive moieties, antibodies, aptamers, folic acid, or drugs [8]. Thus, Pluronic-based nanotechnology is one of the fast-flourishing fields in pharmaceutical research. However, only a handful of Pluronic formulations are in clinical research since these systems show some limitations, mainly their complex characterization, instability under physiological conditions and low loading efficacy. Furthermore, the broad variability of Pluronic polymers requires a thorough study on their toxicity aspects. It has been reported that Pluronics introduced in the body via routes other than dermal exposure have a rapid clearance from the body, thus reducing their potential toxicity. According to the Clinical Toxicology of Commercial Products, most Pluronics are at the lower limit of the “slightly toxic” class, with high values around 5 g/kg [9]. Considering their surfactant nature, the toxicity decreases as the ethylene oxide: propylene oxide (EO):(PO) ratio increases and as the molecular weight of the hydrophobic part increases. In particular, the most widespread member, Pluronic F127, is described as the least toxic member, but can also produce undesirable effects in animals such as hypertriglyceridemia and hypercholesterolemia [9]. Nevertheless, most studies in humans indicate that Pluronics are safe to be used [10].

This review aims to serve as a guide for the reader to understand the parameters involved in the formation of direct as well as reverse Pluronic micelles, ultimately increasing their chances to reach clinical applications. An overview of the most common techniques employed for the characterization of Pluronic micelles is also presented. Finally, we summarize the up-to-date approaches to improve the stability and drug loading of Pluronic micelles. 

## 2. Physicochemical Properties of Pluronics and Self-Assembly Behavior

**Figure 1:**
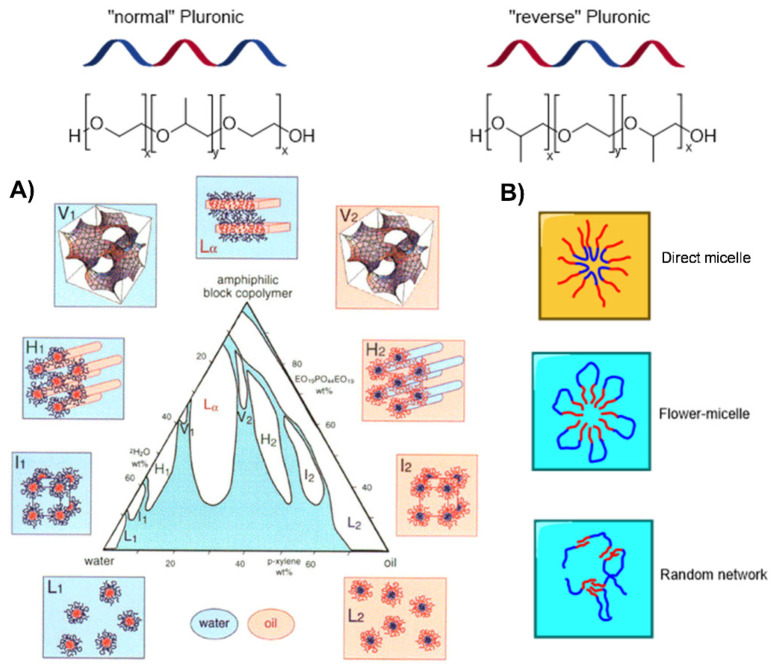
Overview of the different supramolecular structures formed by Pluronic polymers. (**A**) Example of normal P84 in water and *p*-xylene (l_1_, H_1_, V_1_, L_α_, V_2_, H_2_ and l_2_) indicate normal (direct, “oil-in-water”) micellar cubic, normal hexagonal, normal bicontinuous cubic, lamellar, (reverse, “water-in-oil”) bicontinuous cubic, reverse hexagonal and reverse micellar lyotropic liquid crystalline phases, respectively. L_1_ and L_2_ indicate direct micellar and reverse micellar solutions. Reprinted with permission from [22]. Copyright 1998 American Chemical Society. (**B**) Supramolecular assemblies described in the literature for reverse Pluronics, including normal or direct micelles, flower-type micelles and micellar or random networks [23].

Pluronics are synthetic, triblock copolymers composed of ethylene oxide (EO) and propylene oxide (PO) repeating units. The most common copolymers, so-called “normal” Pluronics, exhibit a general formula (PEO)_x_-(PPO)_y_-(PEO)_x_, being hydrophilic poly(ethylene oxide) (PEO) chains on the sides and a central hydrophobic poly(propylene oxide) (PPO) chain. However, reverse analogues (Pluronic-R) are also described with a general formula (PPO)_x_-(PEO)_y_-(PPO)_x_, exhibiting a central hydrophilic chain flanked with two hydrophobic chains (Figure 1). Normal Pluronics are commercially known as Pluronics^®^ (manufactured by BASF, Ludwigshafen, Germany) or Poloxamers^®^ (manufactured by ICI, Tokyo, Japan) while reverse Pluronics are named Pluronics-R (produced by BASF). The nomenclature employed for these polymers—a letter followed by two or three digits—indicates their structural differences and differs depending on the supplier. Poloxamers are named using the letter *P* and three digits: the first two digits represent the molecular weight of the PPO chain, multiplied by 100; the last digit means the PEO percentage in the copolymer multiplied by 10. For Pluronics^®^, the letter indicates the physical state of the product (P = paste; L = liquid; F = flakes); the first and/or middle digits indicate the molecular weight of the PPO block multiplied by 300; the last digit gives the PEO percentage multiplied by 10. For example, Poloxamer P188 or Pluronic L61 is a copolymer build-up with a PPO molecular mass of 1800 g/mol and 80% of PEO. These polymers are non-ionic amphiphiles and exhibit an extraordinary versatility arising from the multiple combinations of molecular weights and commercially available PEO:PPO ratios. These lead to a variety of physicochemical and biological properties, which have been broadly explored for normal Pluronics [11,12,13,14] but less studied for the reverse counterparts [15,16]. 

In solution, Pluronic chains spontaneously form thermodynamically stable supramolecular structures, including micelles, gels, rod-like structures, and lamellar phases (Figure 1) [17]. The polymer composition, concentration and temperature will determine the resultant structure, among other variables. In general, at low concentrations, Pluronic solutions contain individual molecules that aggregate in spherical or rod-like micelles above the CMC, which can further form lyotropic liquid crystals at higher concentrations. Micellization phenomena occurs around the CMC, but above the CMC the micelles may arrange in a lattice structure (gel). The Pluronic concentration at which gelification phenomena occurs is called as critical gel concentration (CGC). The micellization process of Pluronics is entropy-driven and endothermic [18], and it is a less favorable process for reverse Pluronics in water. For normal Pluronics, direct micelles (DMs, in oil in water emulsions) are obtained when a hydrophilic external solvent is used, while reverse micelles (RMs, in water in oil emulsions) are formed in a lipophilic environment [19]. In the past, the term “reverse micelle” was restricted to systems with a low water content, but it is common to use the term for micellar systems in a predominantly organic media [20]. 

These reversible polymeric structures are formed as a result of a delicate balance between strong covalent bonds that hold the PEO and PPO blocks together within each Pluronic unimer and the reversible intermolecular forces that assemble them. The micelles’ size is dictated by different parameters such as the molecular weight of the copolymer, the aggregation number (N_agg_) of the amphiphiles, the ratio of hydrophilic and hydrophobic chains, the amount of solvent trapped inside the micellar core and the preparation process [21].

The micellization process of Pluronics is a spontaneous process that is usually defined by the following parameters: CMC, defined as the concentration at which micelles appear in the solution of an amphiphile at a given temperature, and critical micellar temperature (CMT), as the temperature at which micelles appear in an amphiphile solution of a given concentration. CMC is a critical indicator of micellar stability: the lower the CMC value, the more stable the micelles.

Other parameters include the enthalpy of micellization (ΔH_mic_); the N_agg_, which indicates the number of surfactant unimers in a spherical micelle; and the hydration number, defined as the number of water molecules moving with a micelle as a kinetic entity. The amphiphilic nature of a surfactant, which is crucial for the micellization capacity, is described by the hydrophilic–lipophilic balance (HLB). The HLB is calculated from the weight percentage of the hydrophilic groups to the hydrophobic groups in a molecule, with values ranging from 1–20 [24]. This parameter suggests the type of micelle to be formed: RM for HLB values <10 (predominantly hydrophobic surfactants) and DM for HLB values >10 (predominantly hydrophilic surfactants). The HLB value is also related to the particle size, drug loading, stability, and drug release profile [25]. Considering the HLB value and PPO chain length (“y” value in (PEO)_x_-(PPO)_y_-(PEO)_x_), Pluronics can be divided into four types [2]: type I, “hydrophilic” Pluronics with HLB 20–29, such as F68, F108 and F127; type II, with HLB < 20 and y < 30, such as L35, L44 and L64; type III, with HLB < 20 and y = 30–60, such as P85, P105 and L61; and type IV, with HLB < 20 and y > 60, such as P123 and L121. Polymers with higher HLB values are less prone to form micelles. Taking into account that Pluronic micelles are typically employed as carriers of poorly water-soluble drugs in a water aqueous solution, hydrophobic Pluronics (with low HLB) could improve their loading capacity; nevertheless, these parameters also decrease the colloidal stability of micellar systems, exhibiting high CMC values, which justify that most examples in the literature employ hydrophilic Pluronics.

To characterize the cargo loading within micelles, other parameters are used such as the drug loading degree (LD), the drug encapsulation efficiency (EE), the micelle/water partition coefficient (P) and the standard free energy of solubilization (ΔG^0^). The LD is the mass fraction of the drug in the micelles. The EE is the ratio of the solubilized drug from the original drug feed and is calculated as Equation (1). P indicates the ratio of the drug concentration in the micelle to the drug concentration in water for a particular surfactant concentration (Equation (2)). S_tot_ indicates the total concentration of the drug, while S_w_ indicates the concentration of the drug in water. ΔG^0^ depicts the Gibbs free energy of a transfer of one mole of the drug in the micellar phase (Equation (3)). The more negative the value, the more spontaneous the solubilization.

(1) $$\mathrm{EE}=\frac{\mathrm{mass}\;\mathrm{of}\;\mathrm{drug}\;\left(\mathrm{in}\;\mathrm{micelle}\right)}{\mathrm{mass}\;\mathrm{of}\;\mathrm{drug}\;\left(\mathrm{initial}\;\mathrm{feed}\right)}$$

(2) $$P=\frac{S_{tot}-S_{w}}{S_{w}}$$

(3) $$\Delta G^{0}=-\mathrm{RTlnP}$$

## 3. Factors Affecting the Micellization Process

### 3.1. Pluronic Length, Concentration and PEO:PPO Ratio

**Figure 2:**
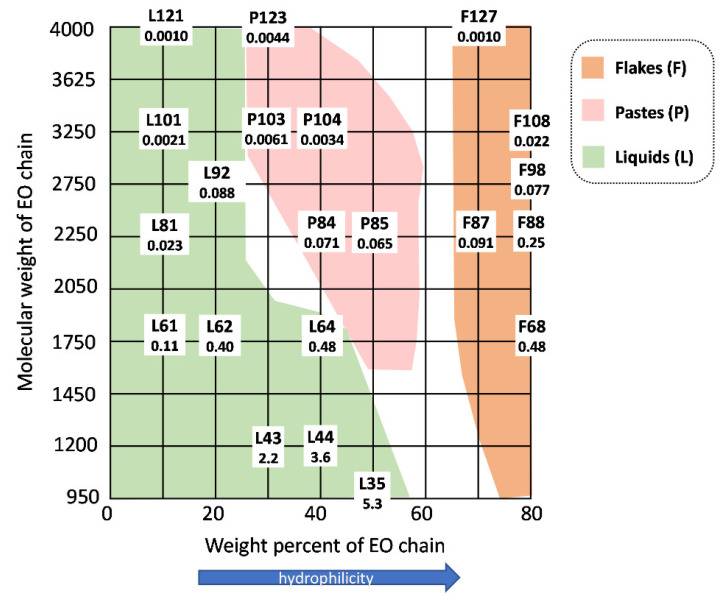
Pluronic grid including CMC values at 37 °C. Adapted from [29,30] with permission from the Royal Society of Chemistry.

In these PEO-PPO-PEO block copolymers, the different composition and length of the blocks lead to varied micelle corona and core sizes. For example, Verma et al. indicated that for Pluronics with a high PEO ratio, water molecules are more liable in the corona of direct micelles, therefore leading to high hydration [26]. Accordingly, both parameters affect the aggregation numbers and determine the solid, paste or liquid nature of the polymer (Figure 2). The PEO:PPO ratio in Pluronics determines the HLB and clearly impacts on the ability of Pluronics to self-aggregate. The adapted Pluronic grid in Figure 2 presents an overview of different polymers comparing the molecular weight of the PEO and PPO chain, and includes the CMC values measured at 37 °C. For example, highly hydrophilic Pluronics (high EO wt%) do not aggregate at room temperature. At a constant PEO:PPO ratio and a given temperature, CMC and CMT decrease when increasing the molecular weight of the polymer. Furthermore, CMT increases when increasing the molecular weight of the PEO or decreasing the molecular weight of the PPO [27], in Table 1. PEO:PPO ratio is also relevant for the stability of the micelles and their compatibility and circulation time in the body. The use of Pluronics with high PEO proportions achieves high stable micelles in water and better compatibility [2]. 

Furthermore, the concentration of Pluronics influences the micellization. The CMT for F127 is reported to decrease with increasing polymer concentration, i.e., from ≈26 °C at 0.40 mM to ≈17 °C at 7.94 mM F127 in water [28]. 

**Table 1:** Selected examples illustrating the impact of the polymer structure on the CMT and CP.

Pluronic	MW (Da)	Molecular Formula	CMT (K)	CP (K)	Ref.
P84	4200	EO_19_PO_43_EO_19_	297 ^a^	346 ^a^	[31]
F87	7700	EO_61_PO_40_EO_61_	301 ^a^	375 ^a^	[31]
F88	11,400	EO_103_PO_39_EO_103_	303 ^a^	375 ^a^	[31]
L64	2900	EO_13_PO_30_EO_13_	305 ^b^	-	[32]
P103	4950	EO_16_PO_56_EO_16_	356 ^b^	-	[32]
P123	5750	EO_20_PO_70_EO_20_	292 ^b^	-	[32]
F127	12,600	EO_106_PO_70_EO_106_	346 ^b^	-	[32]

^a^ Measured at 5% Pluronic concentration. ^b^ Measured at 1% Pluronic concentration.

### 3.2. Normal versus Reverse Pluronics

Beyond the PEO:PPO ratio, the segments distribution within the polymer chain also influences the physicochemical properties of Pluronics. While these properties have been broadly reported in the literature for normal Pluronics, those for reverse Pluronics have been less explored to date. Although reverse Pluronics have a high potential, they present high CMC values that may be unfavorable, as that can affect the stability of micelles. Furthermore, pure vesicles with Pluronic-R have a large size and exhibit phase separation that may affect the efficient loading [23]. Nevertheless, a combination of normal and reverse Pluronics can provide better surface activities and low CMC values, increasing the micelles stability, solubilization capacity and drug loading capacity [33,34]. 

**Figure 3:**
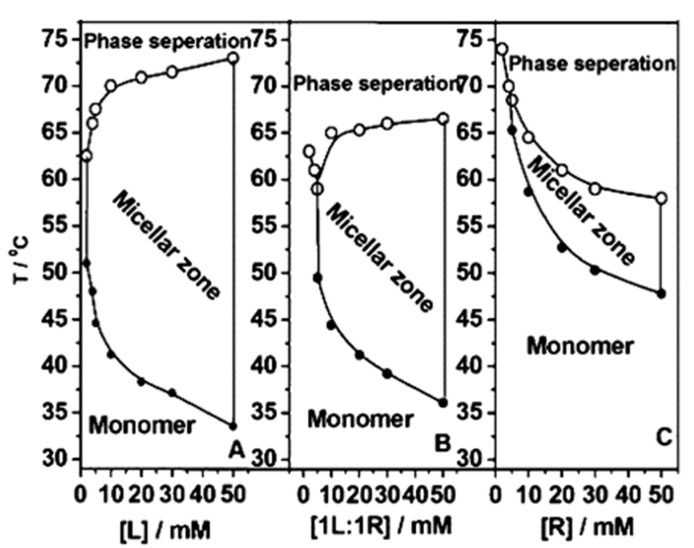
Comparative phase behavior of a normal Pluronic (L44), a reverse Pluronic (10R5) and a 1:1 mixture of both. Reproduced with permission from [15], Copyright American Chemical Society 2012.

Naskar et al. analyzed the behavior in a water solution of normal L44 and reverse 10R5 Pluronics, with a comparable molecular weight of approx. 2000 g/mol [15]. Both formed self-assemblies or micelles, but with prominent differences (Figure 3): L44 presented large polydispersity up to CMT and then became nearly monodisperse at elevated temperatures; however, 10R5 and mixtures L44/10R5 remained polydisperse at all temperatures. Furthermore, 10R5 exhibited a weak biocidal activity towards Gram-positive and Gram-negative bacteria, which was not found for L44. This exemplifies the impact of the fragments’ distribution. It is worth highlighting that reverse Pluronics exhibit a reduced ability to form regular micelles, due to the entropy loss associated with the looping of the middle block. Other aggregates are preferential, such as flower-type micelles and micellar or random networks (Figure 1), with a strong tendency to form structures by sharing one molecule by two micellar cores [23]. For example, 10R5, with short PPO blocks, forms random or micellar networks, while 17R4, with longer PPO blocks, form flower-type micelles. 

Reverse Pluronics can also alter the micellization and gelation of regular Pluronics. For example, the addition of the reverse 25R4 did not alter the thermo-reversibility of the normal F127 due to the constructive interaction between both polymers [16]. However, 25R4 produced a “salt-out like” effect, shifting the F127 micellization to a lower temperature. Under certain conditions, 25R4 also altered the sol-gel transition, acting as bridges to promote the gelification arising from the strong interaction between the PPO blocks of 25R4 chains and F127 cores. Garg et al. evaluated the loading efficacy of Silymarin, an antioxidant drug with poor water solubility, in micellar formulations of normal Pluronics (F108, F127, P85 and P84) with the reverse Pluronic 10R5 [35]. Micelles based on Pluronic mixtures can solubilize appreciable higher amounts of this cargo in comparison to pure micelles. Up to a 491-fold increase of solubility, compared to the aqueous solubility of the drug, was observed with mixed micelles comprising P84:10R5. 

### 3.3. Solvents

Water is a selective solvent for the PEO blocks in Pluronics and the micellization process is less favorable in pure nonpolar solvents. In water solutions, PPO blocks are placed inside the core of micelles protected from the solvent by the outer PEO blocks. For reverse Pluronics, though, micellization in water is a less favorable process considering their different polymeric structure. 

The presence of other solvents alters Pluronics’ behavior and has a pronounced effect on the micellization process (Table 2). For instance, the use of short-chain alcohols such as ethanol provides better solvent conditions for the block copolymers compared to plain water and disfavors the formation of micelles; whereas the use of medium-chain alcohols such as butanol replaces the water molecules in the solvation region of PPO blocks, and thus favors the micellization process [36]. Other solvents, such as *p*-xylene as an example of hydrophobic oil, produce a relevant impact. For example, P104 and L64 led to eight different phases ranging from DMs to RMs in water/xylene mixtures [37,38]. The impact of the percentage of water in the mixture was analyzed using Pluronic F68 or L62 as model polymers [39,40]. Results indicated that the presence of a certain amount of water is required to reach reverse micelles. Sarkar et al. explored the micellization of Pluronics P105 and F127 in binary and ternary solvent mixtures [41]. The binary mixtures consisted of water and one organic solvent (ethanol, glycerol, glucose, propylene carbonate and triacetin), while the ternary mixtures presented water with 50/50 wt% ethanol + glycerol or 50/50 wt% ethanol + propylene carbonate. The authors concluded that glycerol, glucose, propylene carbonate and triacetin promoted micellization through two different mechanisms. Glycerol and glucose dehydrated the PEO–PPO interface and thus reduced the CMC. Propylene carbonate and triacetin located at the PEO–PPO interface and increased its hydrophobicity. In the case of ternary mixtures consisting of water, ethanol (that prevents micelle formation) and glycerol or propylene carbonate (that favor micelle formation), the observed changes in the CMC are subtle. Different self-assembly mechanisms were also detected in P123 micelles in ternary mixtures of water, ethanol and turpentine oil [42]. From several studies, we can conclude that, in general, the addition of (1) glycols maintain the normal micelle stability even when using high glycol/water ratios but do not promote the formation of reverse micelles; (2) oils induce the development of a larger variety of microstructures and reverse structures at high oil/water ratios; (3) solvents such as propylene carbonate, butanol and triacetin have an intermediate behavior between that of glycols and oils. 

The impact of dimethylsulfoxide (DMSO) on the micellization process of Pluronic F127 was studied by Ur-Rehman et al. [43] The mixture of DMSO with water at low concentrations breaks the water channels generated around Pluronic molecules, increasing the hydrophobicity of the structure and reducing the CMT value, and therefore improving micellization. Ionic liquids also promote micellization. The addition of 1.75 M of ethylammonium nitrate (EAN) decreased the CMC to the same extent as a temperature increase from 20 to 24 °C [18]. EAN dehydrates PEO through electrostatic interactions and hydrogen bonding, promoting micellization.

### 3.4. Temperature

It is well reported that temperature alters the thermodynamics of micellization and the structure of Pluronic assemblies. After micellization, water molecules predominantly form hydrogen bonds with the PEO blocks, while the PPO micelle core is mostly dehydrated. At high temperatures, the hydration of the PEO blocks decreases, and the PEO and PPO blocks segregate. The hydrophobic PPO block plays an important role in the assembly of the core-shell micelle structure [13]. Thus, the temperature affects the hydrodynamic diameter of Pluronic micelles. The study showed that the micellar size increased when increasing the temperature for hydrophobic Pluronics such as P84 (which changed from 13.54 nm at 37 °C to 37.54 nm at 57 °C), while the opposite was found for hydrophilic Pluronics such as F127 (it changed from 21.02 nm at 37 °C to 16.89 nm at 57 °C) [44].

In addition, the CMC is strongly temperature-dependent; at a fixed concentration, micellization may be induced by increasing the temperature above a critical value (the CMT). Likewise, the CMT is also concentration dependent. Tsui et al. showed that, at a low Pluronic concentration, the heat of micellization decreases as the temperature increases at the high temperature region [45]. However, at a high concentration, a positive correlation with temperature at the low temperature region was observed.

### 3.5. pH and Ionic Strength

The pH and ionic strength of the solution significantly alter the micellization of Pluronics. For example, a decrease in CMT is found with increasing ionic strength [27]. Considering that the addition of a salt is an easy way to tune the properties of Pluronic solutions, Bahadur et al. studied the impact of inorganic salts (KCNS, KI, KBr, KCl and KF). The influence was explored by measuring the viscosity, the CP, light scattering, pulse gradient spin echo NMR and solubilization assays [46]. The results indicated that these salts have a strong effect on the cloud points of P85 and L64. The polymer chains present a surrounding salt-deficient zone, due to the repulsion of the ions from the poorly polarizable polymer chain. The repulsion is strongest for small, strongly hydrated ions—which may decrease the CP—and least important for large, highly polarizable ions (I^−^, CNS^−^), which increase the CP. The study concluded that the salts influenced the micelle formation, size and shape. Hydrotropes are additives that help solubilizing hydrophobic compounds in aqueous solutions through a different mechanism to micellar solubilization, as they do not self-aggregate. The effect of hydrotropes sodium benzene sulfonate (NaBS), sodium toluene sulfonate (NaTS) and sodium xylene sulfonate (NaXS) on the micellization of L62 in an aqueous solution was studied [47]. The addition of hydrotropes increased the CMC of L62, by enhancing the solubility of PPO moiety (and PEO).

### 3.6. Nature of the Encapsulated Cargo

Pluronic micelles efficiently encapsulate hydrophobic payloads, including drugs, essential oils and antioxidant molecules. The size of the hydrophobic blocks and the nature of the substituents present in the core mainly control the drug loading capacity of polymeric micelles. In general, these nanosystems substantially increase the solubilization of low polarity substances, whereas the increase is rather small for more polar substances [48]. The nature of the cargo also modulates the micelle properties by, for example, producing an abrupt decrease in the CMT of the pristine micelle. It was observed that highly lipophilic drugs increased the sizes of the micellar structure (core and corona) while generally decreasing the micellar aggregation numbers [49].

**Figure 4:**
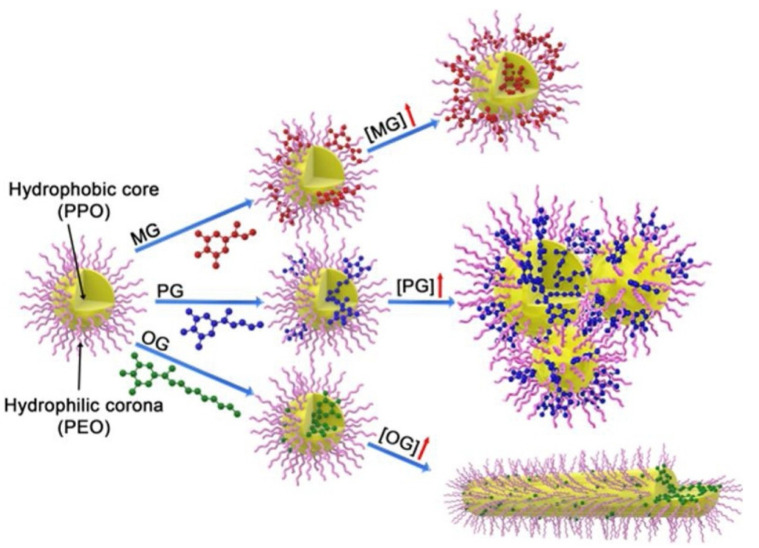
Impact of different gallate derivatives on the morphology of Pluronic P123 micelle. Reprinted from [51], Copyright 2019, with permission from Elsevier.

Highly hydrophobic but non-ionizable molecules, such as essential oils, favored the formation of micelles [50]. The resultant NPs appear as an almost purely oily core surrounded by a first shell composed mostly of PPO and few percent of oil, stabilized in water by the highly hydrated PEO layer. Antioxidants, such as methyl gallate (MG), propyl gallate (PG) and octyl gallate (OG), were encapsulated in P123 micelles. The micelles’ morphology strongly depended on the concentration and alkyl chain length of the gallate (Figure 4). MG molecules are distributed in both the interior and external part of the P123 aggregate and some of them exist in the water solution. PG molecules are mainly located at the interface between PEO and PPO blocks, while a small amount of them is still dispersed in water. OG molecules are completely encapsulated into the hydrophobic PPO cores of aggregates due to their higher hydrophobicity. Cryogenic transmission electron microscopy (cryo-TEM) revealed that spherical P123 micelles form micellar clusters in the presence of PG and short rod micelles with OG, while MG does not change the micellar morphology.

Other cargo, such as cholesterol, have been demonstrated to abruptly change the micelle’s structure. For example, with the addition of cholesterol, F127 micelles spontaneously form niosomes [52]. A dynamic light scattering (DLS) study confirmed the change of size from 27 nm to 100–500 nm diameter, and Förster resonance energy transfer (FRET) assays measured different donor–acceptor distances during this transition, indicating that they are located in different regions of the assemblies. 

Ionizable cargo can generate pH-responsive nanocarriers, which can modulate the release of the drug. Pluronic F127 micelles were used to encapsulate aspirin, erythromycin and ibuprofen [49]. The results indicate that more hydrophobic drugs ensure a more compact packing within the micellar cores at temperatures above 40 °C. On the other hand, an increase in the solution pH leads to ionized drugs in the micellar core and enhanced repulsive interactions that increase the micelles’ size. For pH > 11, fully ionized drugs are released from the micellar cores to the aqueous medium. A similar behavior was observed with flurbiprofen [53]: below the pK_a_ of the drug (6.5), the drug promotes micellization due to hydrophobic interactions between the PPO and the drug; for pH values above pK_a_, the drug is ionized and the release from the core becomes enthalpically and entropically favorable.

## 4. Techniques to Characterize the Micellization Process

A thorough characterization of the structure and properties of Pluronic micelles is fundamental to understand their biological behavior [54]. In this section, we present an overview of the main parameters and techniques described in the literature, including relevant examples. Table 3 and Table 4 summarize relevant information regarding micelles detection and characterization.

### 4.1. Scattering Methods 

**Small-angle X-ray scattering (SAXS) and Small-angle neutron scattering (SANS)**. SAXS and SANS are complementary techniques which enable the study of structures with sizes on the Angstrom scale (tens to hundreds). They are based on the same principles (radiation interacts with a sample and generates a scattering pattern), but SAXS scattering is based on electron densities, while the SANS scattering is based on the differences of the nature of the atomic nucleus. The scattering pattern provides information on the sample shape and aggregation number, and it is especially useful to study non-spherical micelles or heterogeneities in the sample (molecular assemblies, pores or voids) [55,56]. In the case of Pluronic micelles, SAXS provides a full description of the micelles while SANS is mostly sensitive to the hydrophobic core, and the highly hydrated shell is almost invisible. However, they are complementary as the scattering length density (SLD) is significantly different for X-rays (sensitive to electron density, EO > PO > water) and neutrons (sensitive to H/D isotopic composition, deuterated solvent >> EO > PO). Manet et al. employed SAXS–SANS to provide a detailed description of P123 micelles using a simple core–shell spherical model, including their size, shape, aggregation number and detailed composition [57]. The impact of temperature, pH, acid source and swelling agents was also described. A similar study was performed on Pluronic F127 micelles used as carriers of essential oils [50]. At 37 °C, the analysis demonstrated a partial phase separation between the oil and the PPO core, while the PEO shell remained unchanged. RMs have been also characterized through these techniques [58]. 

**Dynamic light scattering (DLS) and Static light scattering (SLS)**. DLS is a non-invasive technique where the sample is exposed to a monochromatic wave of light, generating a light scattering that depends on the size and shape of the molecule [59]. DLS has been widely used to characterize micelle formation as it provides information about the size and various hydrodynamic and thermodynamic properties of micelles in a solution. It is ideal for studying micelles, especially for spherical-shaped particles, and more concretely, those with narrow distributions [56]. DLS also estimates the width of the distribution by the polydispersity index (PdI). Polydispersity can occur due to the size distribution in a sample, or agglomeration or aggregation of the sample, during isolation or analysis. PdI values range from 0.0 (for a perfectly uniform sample with respect to the particle size) to 1.0 (for a highly polydisperse sample with multiple particle size populations), which is considered a good PdI value for a homogenous population change with the type of particles studied. In all cases, values greater than 0.7 indicate that the sample has a very broad size distribution and is probably not suitable for DLS. The combination of size and PdI values give information about the presence of micelles (Table 3).

**Table 3:** Micelle detection chart, considering the size and PdI of the aggregates [59,60].

Size (nm)	PdI		
	<0.50	0.50–0.70	>0.70
<2	No aggregates.	No aggregates.	No aggregates.
2–10	Small monodisperse aggregates. Not compatible with micelles	Small polydisperse aggregates. Not compatible with micelles.	Very polydisperse aggregates. Not compatible with micelles.
10–100	Monodisperse aggregates. Compatible with micelles.	Polydisperse aggregates. Compatible with micelles.	Very polydisperse aggregates. Not compatible with micelles.
>100	Big monodisperse aggregates. Not compatible with micelles	Big polydisperse aggregates. Not compatible with micelles.	Very polydisperse aggregates. Not compatible with micelles.

In SLS, the laser light is directed at the sample and the scattered light is measured at one or more angles around the molecule or particle in a solution. It is also a non-invasive technique that shows the same experimental setup but a different light source than SAXS and SANS, where the sample is subject to a neutron or X-ray, respectively, instead of light. 

DLS and SLS can help to determine the CMC, as there is an increase of scattered light when the CMC is reached [61]; however, DLS needs more time than SLS to detect when the aggregations begin. Nevertheless, this technique is preferred to compare sizes from day-to-day or batch-to-batch, and for measuring how the size of your particles change over long isothermal experiments. Thapa et al. studied the CMC of curcumin-loaded micelles of Pluronic F127 by DLS amongst other techniques [62]. They found two major size-distribution peaks at 4–8 nm and 20–150 nm, assigned to unimers and micelle/micelle aggregates, respectively. Both DLS and SLS were used to characterize the temperature-induced micellization of Poloxamer 188, finding three distinctive regions: unimer, transition and micelle [63].

### 4.2. Spectroscopic Methods

**Infrared (IR) spectroscopy and Raman spectroscopy**. IR and Raman spectroscopies are complementary techniques. They are based on the absorption of radiation by the molecules. The photon energies induce vibrational excitation, which can cause a change in the dipole moment. The vibration of bonds includes stretching, scissoring, bending, twisting and rocking. Interactions between molecules (hydrogen bonding, van der Waals, etc.) can also be identified in IR spectra. 

Sturcová et al. employed IR, in particular attenuated total reflectance–Fourier-transform infrared spectroscopy (ATR-FTIR) and Raman, together with Density functional theory (DFT) calculations, to gain insight on the role of hydration and water coordination in the formation of Pluronic micelles [64]. They showed that hydroxyl stretching offers relevant information about the restructuring of water and changes of polymer chains in polymer/water aggregates. This study relates nanoscale phenomena (hydrophobic and hydrophilic hydration) with the mesoscale phenomenon of micellization. The IR bands of Pluronic methyl groups can also offer valuable information. In water solutions, when the temperature approaches the CMT, the antisymmetric C-H stretching vibration of methyl groups shifts towards a lower wavenumber, as an indication of a progressively less polar environment [65]. An increase of temperature produces a higher proportion of dehydrated methyl groups, arising from a denser PPO core in the micelles. IR is especially useful to study reverse micelles. It can quantify the water content of reverse micelles through the intensity of the OH band at 3420 cm^−1^, as well as visualize the interaction between the micelle and a target molecule, such as a drug [66]. 

**Nuclear magnetic resonance spectroscopy (NMR).** NMR is a spectroscopic technique where the sample is exposed to a magnetic field that produces the excitation of the atomic nuclei (spin). This excitation depends on the intramolecular magnetic field around the atom, so NMR is usually used to identify the electronic and molecular structure of compounds. NMR has been widely used in colloid science, considering that spin interactions change when the polymer is forming aggregates as well as when the solution is isotropic (micelles) or anisotropic. Hence, NMR is used to establish phase diagrams [67] and determinate the molecular interactions between the solubilized drug and the Pluronic micelles. 

Ma et al. used ^1^H-NMR spectroscopy to characterize the interaction of urea with different Pluronics [68]. The study demonstrated that urea interacts selectively with the PEO blocks and stabilizes the gauche conformation of the PPO block, thus increasing the CMT. However, this interaction does not depend on temperature. Foster et al. studied the effect of ibuprofen addition and pH variation on Pluronic P104 solutions through pulsed-field gradient NMR [69]. The addition of ibuprofen to the solution induced the micelle formation, observing a strong pH dependence, as at a pH above the pK_a_ of ibuprofen, it was released from the micelles, leading to a reduction in the polymer aggregation. NMR can be used to determine the CMC in flurbiprofen-loaded DMs [53,70]. 

**Ultraviolet-visible spectroscopy (UV-Vis).** UV-Vis spectroscopy relies on the absorption of UV-Vis radiation by the molecules, undergoing electronic transitions from the ground state to the excited state. In colloid science, UV-Vis spectroscopy is used to study the CMC of amphiphilic compounds at different conditions. In a typical assay, the UV-Vis absorption of a probe such as fluorescein or pyrene is studied: above the CMC, the probe in entrapped in the micelles and the UV-Vis absorption pattern changes [71]. This technique is also used to evaluate a drug’s solubilization in micelle structures and the micellar/solvent partition coefficient, helping to determinate the interaction between the drug and the Pluronic chains and the location of the drug in the micelle. Singla et al. measured the oxcarbazepine solubility and its micelle/water partition coefficient in F108, F127 and P84 micelles [72]. In addition, UV-Vis spectroscopy was employed by Kadam et al. to evaluate the impact of the Pluronic type, temperature and salt presence on the carbamazepine solubility in the micelles [73]. Thapa et al. studied the impact of additives such as ethanol or DMSO on the solubilization of curcumin in F127 micelles [62]. UV-Vis, DLS and DSC confirmed that curcumin significantly enhanced F127 micellization, while the effects of ethanol and DMSO were lower. On the other hand, the solubility of different antirheumatic drugs in F127 in a buffer solution was tested [74].

**Fluorescence spectroscopy.** Fluorescence spectroscopy is based on the excitation of electrons in a molecule when they are hit by a beam of light, usually UV light. This excited state is followed by rapid thermal energy loss, producing the emission of a photon that is detected. Some molecules and materials are intrinsically fluorescent, but non-fluorescent systems can also be studied by adding a fluorescent tag or probe. In colloid science, fluorescence spectroscopy can be used to study the CMC of amphiphilic compounds at different conditions. Lee et al. studied the micellization of Poloxamer 407 in water and its dependence on temperature using six different fluorescent probes [75]. In the case of mixed micelles formed from hydrophobic L81 and hydrophilic P123, pyrene fluorescence spectroscopy confirmed that the CMC was 0.032 mg/mL, located in between that of the individual Pluronics [76]. On the other hand, Basak et al. analyzed the hydration of the Pluronic F127 micellar core loaded with ibuprofen or aspirin, observing that low temperatures promote the entrance of the solvent in the micellar core raising the micellar size, while the solvent is excluded from the micellar core at high temperatures causing a more hydrophobic core and compact structure [49].

### 4.3. Rheological Methods

**Viscoelastic behavior**. The viscosity measures the internal resistance to flow of a material. A perfect case of viscosity follows Newton’s law, which states that shear stress is directly proportional to velocity gradient. Lots of compounds such as water or some oils follow this law, but many systems such as polymers, colloids and gels are non-Newtonian. For example, rheological properties of F68 micelles are modified depending on the type of binary (Pluronic/water and Pluronic/organic solvent) and ternary systems (Pluronic/organic solvent/water) formed, reflecting the changes of the F68 solution behavior. In particular, high relative viscosity was observed because of the growth of the micelles’ size, resulting from the successive shape transformation from the micelles to the ellipsoidal or worm-like structures. Viscoelastic behavior can also be used to identify the CMC of colloid systems. Kurumada et al. studied the CMC of Pluronic F127 at different concentrations and temperatures in an aqueous solution by viscosity measurements, obtaining values of 1.0 g/L in the region of 20–50 °C [77]. Costanzo et al. employed SAXS and rheological studies to develop a consistent link between the microstructural evolution of Pluronic F68 and the macroscopic flow properties [78]. They observed a transition from the viscoelastic solution state—from a disordered micellar structure—into a soft solid, assigned to soft body-centered cubic crystals. 

### 4.4. Microscopy-Based Methods

**Transmission electron microscopy (TEM).** TEM is based on the irradiation of a thin sample with a 200 keV electron beam. Some of these electrons are transmitted, some of them are dispersed and some of them give rise to interactions that produce different phenomena such as light emission, Auger emission, X-rays, etc. The transmission electron microscope uses electron transmission/scattering to form images, electron diffraction to obtain information about the characteristic crystal structure and X-ray emission to know the elemental composition of the sample. This technique can be used to identify and measure micelle formations [79]. TEM measurements require a high-vacuum and thin layer of specimen for the penetration of the electron-beam through the sample, which may affect the physicochemical conditions of the sample. Thus, the cryo-TEM method is recommended, as it does not require any staining or fixation of the sample in the native environment [80]. The sample is examined at the cryogenic temperatures (usually liquid-nitrogen temperatures). Pluronic micelles in water can be clearly visualized by cryo-TEM, achieving a detailed inspection of the individual micelles, their size, shape, core–shell structure as well as long-range order [81]. 

**Freeze-fracture electron microscopy (FFEM)**. FFEM is a method that consists of rapidly freezing a sample to break it (fracturing) to expose a fractured plane. This plane is then visualized by electron microscopy. FFEM can be employed to measure micelle aggregates [82]. 

**Atomic force microscopy (AFM)**. AFM is a high-resolution technique (up to 0.1 nm) that provides useful information about the morphology and size of different nanosystems, including micelles [83]. The sample is deposited as a thin layer on a support and then scanned with a sharp tip probe determining the topography of the sample. The sample must be strongly attached to the support in order to resist the forces exerted by the scanning tip, which may disrupt the micelle. Curcumin-loaded F127 and F68 micelles were examined through AFM and their spherical shape was confirmed with diameters below 100 nm [84]. 

### 4.5. Calorimetric Methods

**Isothermal titration calorimetry (ITC)**. ITC is a powerful and very sensitive thermodynamic technique used to study binding interactions between molecules in dilute aqueous solutions through absorbed or released heat. It provides information regarding the affinity, the enthalpy and the stoichiometry of the reaction. Naskar et al. determined the CMC, CMT and CP of both normal L44 and reverse 10R5 Pluronics in water by ITC, together with DLS and spectrophotometry, Figure 3 [15]. In another study, Prasanthan and Kishore determined the CMC of Pluronic F127 and F68 micelles loaded with the chemotherapeutic drugs gemcitabine, cytarabine and hydroxyurea and its interaction with the protein albumin [85]. The combination of ITC and SANS measurements confirmed that the formation of P85/P123 mixed micelles, whose properties, including size, CMC, and enthalpy of micellization, depend on the composition of Pluronic solutions [86]. 

**Differential scanning calorimetry (DSC**). DSC determines the amount of heat absorbed or released by a sample when a perturbation caused by a change in temperature occurs [87]. This energy is known as enthalpy (at constant pressure). DSC measures the heat flux (mJ/s) between the sample and an inert reference, i.e., the thermal transitions. DSC is usually used for determining the alterations in structural properties of a sample as a function of time and temperature, but it is also a useful technique to study micellization processes [88]. In order to determine the CMT from a thermogram, three methods can be employed: the onset temperature of the peak (T_onset_), the inflection point temperature (T_inf_) and the peak maximum temperature (T_max_). T_max_ is the preferred system as it is less influenced by the polydispersity of the polymers. Thapa et al. studied the CMT of F127 micelles in the presence of curcumin [62]. The thermograms showed a broad endothermic peak with a visible shoulder. The peak with the lowest T_max1_ was ascribed to the organization of monomers into aggregates while T_max2_ was identified as the CMT. Lee et al. studied the CMT of heparin-loaded micelles of Pluronic F127, indicating that heparin promoted micellization, as confirmed by the decrease in CMT [89].

### 4.6. Other Methods

**Solubilization measures**. Micelles can solubilize hydrophobic compounds in hydrophilic media (direct micelles) or hydrophilic substances in lipophilic media (reverse micelles). Micellization can be studied through the solubilization of such compounds as there is a measurement discontinuity when micelles are formed. For example, Zhang et al. studied the micellization of Pluronic P85 by analyzing the ability to encapsulate decane in a polyethylene glycol (PEG) 200 solution [82].

**Zeta potential (ZP).** ZP can be defined as the magnitude of the overall charge acquired by any particle in a particular medium and is a common parameter used to measure the stability of colloidal systems. ZP has a direct correlation with the electric repulsion or steric barriers between particles. Thus, a low ZP indicates low stability, as the system will end aggregating, while a high ZP is related to high stability of the system. Generally, stable nanocarrier dispersions exhibit ZPs higher than +30 mV or lower than −30mV [80]. The stability of different Pluronic micelles loaded with poorly water-soluble anticancer drugs was confirmed through the ZP [90]. In addition, the ZP value may also provide information regarding the blood circulation times of a nanocarrier, its cell permeability and biocompatibility. Several ZP instruments may be used, based on electrophoresis or electroacoustic approaches. 

**Surface tension.** The surface tension of a solution can be defined as a property of the surface that allows it to resist external forces. In a liquid, molecules share cohesive forces between them, but in the surface, there are no molecules above, so the cohesive forces there are stronger than in the rest of the liquid. When micelles form in a solution, a break in the surface tension happens, thus enabling the study of the CMC. Alexander et al. studied the CMC of P103, P123 and L43 by surface tension measurements at different pH levels [53], while Bak et al. explored the influence of F68 in perfluorodecalin-containing mixtures [91].

**Table 4:** Relevant parameters for Pluronic micelles and characterization techniques to measure such parameters.

Parameter	Technique	Example
Morphology and size (mean, distribution profile, hydrodynamic radius, PdI, hydration)	TEM CryoTEM FFEM DLS/SLS SAXS/SANS AFM IR/Raman Fluorescence spectroscopy	Doxorubicin-loaded DMs [79] Pristine DMs [81], Gallate-loaded DMs [51] DMs aggregates [82]. Pristine DMs [63] Lamotrigine-load DMs [44] DMs [57], RMs [58], oil-loaded DMs [50] Curcumin-loaded DMs [84] DMs [64,65], RMs [66] Ibuprofen and aspirin DMs [49].
Aggregation number, cloud point	ITC SANS	Lavender-oil loaded mixed micelles [86] Lamotrigine-loaded DMs [44]
CMC	DLS Surface tension NMR UV-Vis spectroscopy Fluorescence spectr. Cyclic voltammetry Rheology (viscoelasticity) Electrical conductivity ITC	Curcumin-loaded DMs [62] DMs [91] Flurbiprofen-loaded DMs [53,70] Curcumin-loaded DMs [62] Mixed micelles [76] DMs [75,92] DMs [77,78], RMs [39] Neutral-ionic mixed DMs [93] Gemcitabine, cytarabine-loaded DMs [85], Pristine DMs and RMs [15]
CMT	ITC DSC	Pristine DMs and RMs [15] Heparin-DMs [89], curcumin-DMs
Thermodynamics (enthalpy and entropy of micellization)	ITC	DMs [18]
Integrity and stability in solvents or relevant media	ZP FRET	Camptothecin-loaded DM [90] Cholesterol-induced DM-noisome transition [52]
Cargo loading - Capacity - Locus - Efficiency - Partition coefficient, free energy of solubilization - Interactions with cargo	UV-Vis spectroscopy NMR/ITC Equation (1) Equations (2) and (3) NMR IR	Oxcarbazepine DMs [72] Flurbiprofen-loaded DMs [53,70] Curcumin-loaded DMs [94] Ciprofloxacin-loaded mixed micelles [95] Gallate-DMs [51], Ibuprofen-DMs [69] RMs [66]
Cargo release (in solvents or relevant media)	Dialysis	Oxcarbazepine DMs [72]

## 5. The Lights and Shadows of Pluronic Micelles in Drug Delivery 

Pluronic micelles have been extensively explored as drug delivery agents towards diverse pharmaceutical and biomedical applications [2,7,11]. In addition to increasing the drug solubility, they present many advantages, including long blood circulation time, strong cellular uptake as well as low cytotoxicity. Micelles can improve the drug bioavailability by prolonging the circulation time of the encapsulated drug and slowing down the dissociation of drugs from the micelles in the blood circulation system [96]. Furthermore, they can generate an efficient drug accumulation at the target site through the enhanced permeation and retention (EPR) effect. Consequently, they have been broadly explored in several fields of biomedicine [29], such as cancer [2] and antimicrobial therapy [97]. Most studies focus on human applications, but nanotechnology-based pharmaceutical products can also be applied to veterinary pharmacotherapy [98]. 

Despite the verified advantages, Pluronic micelles face some drawbacks. On the one hand, stability issues may arise in the physiological environment. When a micelle solution is diluted to a very low concentration (<10^−3^ mM), the surfactant content is not sufficient to drive the self-assembly of micelles. They also undergo environmental changes, including exposure to pH changes, and contact with numerous proteins, lipids and cells. Micelles tend to dissociate and prematurely release the cargo, decreasing the efficacy and producing toxicity concerns. In addition, some pharmaceutical formulations need to avoid the presence of water due to the instability of APIs. On the other hand, micelle systems may include low drug concentrations, thus limiting the use of this nanotechnology to drugs with a high therapeutic potency. Thus, it is necessary to improve the drug loading efficiency to extend the implementation of this type of nanosystem. 

This section focuses on presenting state-of-the-art approaches to overcome these drawbacks, in order to offer useful alternatives to traditional strategies that may facilitate the transfer of Pluronic micelles from the bench to the clinic. 

### 5.1. Approaches towards an Increased Stability of the Micelles

Beyond simple strategies, such as precisely controlling the storage conditions of the micelles, the fundamental strategy to improve the stability of micelles is to enhance intra-micellar interactions. A high self-assembling potential promotes the use of a lesser amount of Pluronic to form the micelles, observing a low CMC value. Thus, CMC may be used as an indicator of micellar stability, with an inverse correlation between both variables. Several approaches are described in the literature to decrease the CMC value, as described below. 

**Structural design of polymer precursors.** A wise design of the polymer, such as altering the polymer length, the hydrophilic/hydrophobic block ratios or increasing the crystallinity of hydrophobic segments, can improve the micelle’s stability. For example, the CMC values of Pluronics range from 5.3 mM for L35 to 0.0010 mM for L121, being the later more stable (Figure 2). With the increase in the size of the hydrophobic block, the CMC value drops exponentially. It is worth highlighting that (1) the HLB value and PPO chain length of Pluronics are an indicator of micellization, but they are also connected to the micelle’s stability [2]. In fact, type I and II Pluronics can be used as a nanostabilizer to prevent aggregation issues and promote more stable structures; (2) reverse Pluronics show higher CMC than normal Pluronics promoting unfavorable micelle structures in terms of stability, in addition to forming systems with a large size and high risk of separation [35].

Traditional block copolymers have been also upgraded to generate amphiphilic polymeric structures with lower CMC and higher drug loading [99]. For example, random, graft, alternate and starshaped polymers are under study, and it is also a current trend in the field of Pluronic derivatives. 

**Solvent mixtures.** As previously mentioned, the impact of the solvent in the micellization process is outstanding and can be employed to increase the stability of the aggregates. For example, a different self-assembly mechanism was observed for P123 micelles in pure solvents and in ternary mixtures of water/ethanol/turpentine oil. As Zhao et al. described, the addition of 1.5% turpentine oil in the solution with 5% Pluronic P123 and ethanol–water solvent (25:100) helps to swell the core of the micelles promoting the transformation of cylindrical to spherical micelles, which are more stable systems [42]. 

**Figure 5:**
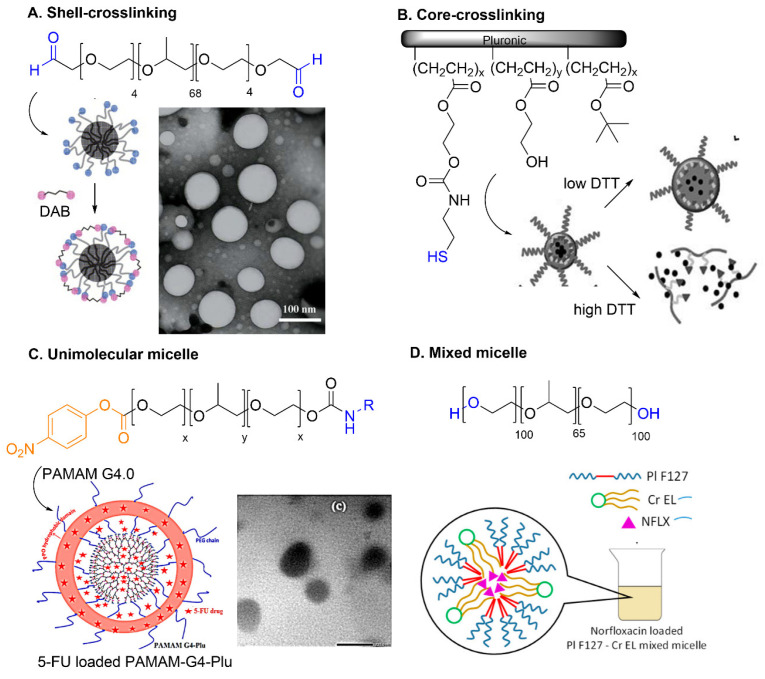
Diverse strategies to improve micelle stability. (**A**) Shell-crosslinking. Reprinted from [100], Copyright 2007, with permission from Elsevier. (**B**) Core-crosslinking. Reproduced from [102]. (**C**) Unimolecular micelle. Reprinted from [48], Copyright 2017, with permission from Elsevier. (**D**) Mixed micelle. Reprinted from [104].

**Shell or core crosslinking.** Micelle formation is reversible and highly dependent on weak intermolecular interactions within the micelles. The crosslinking of specific domains, such as the shell or the core, stabilizes the micelles [4]. Typical crosslinking methods include the polymerization of radicals, addition of bifunctional crosslinkers or click chemistry. For example, Yang et al. developed shell-crosslinked Pluronic L121 micelles through reversible Schiff base bonds [100]. The resultant spherical micelles presented particle sizes around 100 nm (Figure 5A). After crosslinking, the micelles exhibited a 10-fold lower CMC, which indicates a 10-fold more volume of water to dissociate the micelles into unimers. Shell-crosslinking of F127 micelles also decreases the premature release of combretastatin A4 loaded in the nanocarrier [101]. Core-crosslinking is a more desirable approach, avoiding potential changes on the micellar surface. Abdullah et al. designed core-crosslinked Pluronic nanoparticles through reversible disulfide bond formation (Figure 5B) [102]. Thiol-decorated F127 led to micelles with around 100 nm diameter, which increased to around 235 nm after the encapsulation of paclitaxel. These micelles showed effective stability in blood–plasma conditions and disassembled in the presence of higher concentrations of dithiothreitol. 

**Unimolecular micelles.** Unimolecular micelles are an extraordinary approach to increasing stability without crosslinking steps. This stability arises from their own structure, comprising a single molecule—a multi-arm star amphiphilic block copolymer or a telodendrimer. Different Pluronics were attached to the periphery of a fourth generation PAMAM dendrimer, generating micelles with diameters of around 60–180 nm (Figure 5C) [48]. The nanocarriers exhibited a high drug loading efficiency (up to 76%) of fluorouracil and a high anti-proliferative activity against the MCF-7 breast cancer cell. Other unimolecular micelles from PAMAM-F127 were prepared and loaded with doxorubicin (DOX). A 33-fold increase in cytotoxicity, a much quicker uptake and 5-fold accumulation of conjugates were observed compared with free DOX [103].

**Pluronic-based mixed micelles.** The inclusion of other surfactants may improve the stability and drug loading efficiency of Pluronic micelles. Nambam et al. analyzed the use of ionic and non-ionic surfactants as additives in Pluronic F108 systems, concluding that the use of anionic sodium dodecyl sulfate promotes the formation of a strong electrostatic barrier which minimizes the self-assembly and the growth of micelles [105]. Gao et al. reported that micelles that combine Pluronic P105 with d-alpha-tocopheryl polyethylene glycol 1000 succinate (PTGS) at molar ratio 3:7 show low CMC value, therefore forming more stable systems, thanks to the presence of an aromatic ring of TPGS which increases the hydrophobic interactions between the polymer chains in the micelle’s core [106]. Mixed micelles based on two nonionic surfactants, Pluronic F127 and cremophor, were designed [104] (Figure 5D) and the interactions between the two surfactants was studied through the parameter β proposed by Rubingh [107]. This parameter is negative if synergism is present, leading to the formation of micelles at a lower concentration than the pure surfactants alone. In this study, a moderate synergistic effect was observed, decreasing the CMC from 80 µM to 28 µM. Many other recent studies exemplify the usefulness of mixed micelles based on Pluronics and other components such as labrasol [108], the cleavable cationic surfactant 16-E1–16 [109], phosphatidylcholine [110] or mixed Pluronics [111,112,113]. 

### 5.2. Approaches towards an Improved Drug Loading in the Micelles 

Drugs are embedded in the core–shell structure of the micelle. The loading efficiency depends on the initial amount of drug added, the aggregation number of the polymer and the miscibility between polymers and drugs, which is mainly related to the extent of the hydrophobic interaction between the drug and the micellar core. For example, Kaur et al. reported that the loading efficiency of quercetin and curcumin varies depending on the type of Pluronics used (P84, F68 or F127) and the labrasol inclusion [108]. Water-insoluble drugs can be encapsulated in the micellar core by physical entrapment, where the drug is included into the hydrophobic core during or after self-assembly by dialysis and oil-in-water emulsion, but chemical linking may be used where the drug is linked to the hydrophobic block before self-assembly. Physical connections are often preferred, as higher drug loading is possible without drug modifications, but this section focuses on describing alternative approaches to increase the loading efficiency. 

**Pluronic-based RMs.** RMs result from the spontaneous self-assembly of a surfactant in non-polar solvents [114]. In the presence of water or other polar solvents, RM aggregates are taken up inside the hydrophilic internal region of the two-component cluster, thereby forming isolated droplets uniformly dispersed in the bulk solvent (“swollen micelles”). RMs present an extremely important difference compared to other types of microemulsion; they are optically transparent solutions and have a recognized homogeneity, monodispersity and stability. Furthermore, they present thermodynamic stability, spontaneous formation, large surface area (10^2^–10^3^ m^2^·cm^−3^), low interfacial tension (<10^−2^ mN·m^−1^), nanometer size (<100 nm) and a highly dynamic nature. RMs are useful carriers for poorly water-soluble drugs. Different examples of Pluronic-based RMs have been described along this review [39,40,58,66], which illustrate the potential of these nanocarriers.

**Pluronic-based mixed micelles.** Mixed micelles are a promising approach to increase not only the stability of the micelles, but also the drug loading. Considering that most micelle-based pharmaceutical systems include actives with poor aqueous solubility, their loading efficiency is directly proportional to the hydrophobic core of DMs. Thus, using Pluronic mixtures with different hydrophilia balance may have a positive impact on both parameters. By adding a small percentage of the hydrophobic Pluronic L101 to P105 micelles loaded with paclitaxel, the drug-loading coefficient increased from 1.0% to 1.7% and the encapsulation ratio from 54.5% to 83.3% [115]. Similar effects on the drug loading is observed for the drug ciprofloxacin if combined F127 and L64 [95]; aceclofenac in a mixed Pluronic combination with L81 and F108 [111]; and quercetin in F123/F88 systems [110]. Pellosi et al. compared the stability and the loading capacity of P123 and F127 micelles with mixed P123/F127 micelles, with benzoporphyrin derivatives obtaining a higher stability and loading capacity with the mixed micelles [116]. On the other hand, the use of a binary mixture of reverse Pluronics with normal Pluronics can also enhance the drug loading. Garg et al. observed that the mixing of reverse Pluronic 10R5 with normal Pluronics (F108, F127, P85 and P84) can solubilize and load appreciably higher amounts of the hydrophobic drug silymarin [35]. Carvedilol is a hypertensive drug whose aqueous solubility increased up to 271-fold as a result of its encapsulation within a mixed micelle formulation based on Pluronic^®^ F127, D-α-tocopheryl polyethylene glycol 1000 succinate (PTGS) and L-cysteine HCl [117].

**Figure 6:**
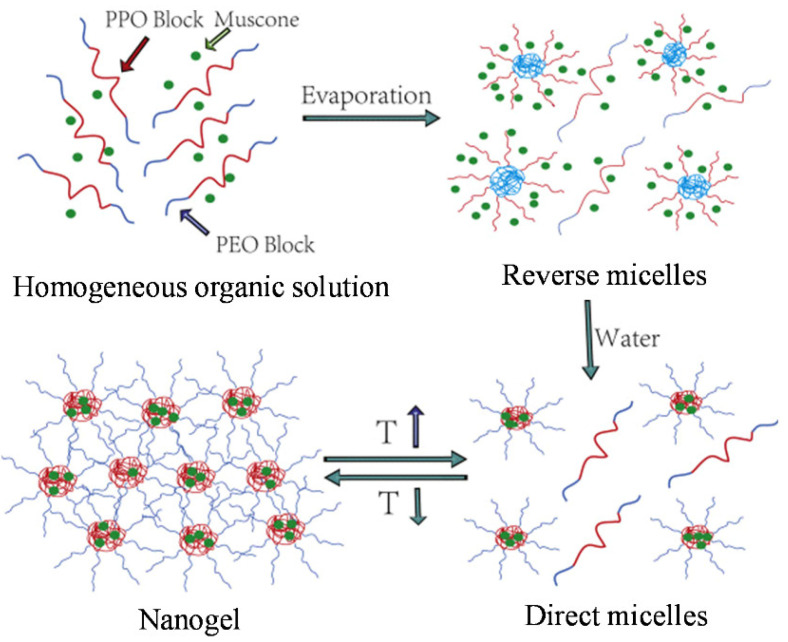
Design of muscone thermoresponsive nanogels prepared by RM → PM method. Reprinted from [118], Copyright 2017, with permission from Elsevier.

**Pluronic nanogels and hydrogels**. The self-assembly of nanosized drug delivery systems can further enhance the uniformity and solubility of poorly water-soluble drugs. Through the “reverse micelle to positive micelle” method of Pluronic P407 (Figure 6), Wang et al. designed an ophthalmic delivery system of muscone based on thermoresponsive nanogels [118]. These RM nanogels showed a more uniform, narrow particle size distribution by DLS (in the range 0.086–0.122 PdI and 56.4–90.8 nm) and a higher muscone loading (98–100% EE) than nanogels obtained through traditional approaches (PdI 0.453, 354.3 nm and 14.7% EE). The dispersion of drug-loaded nanocarriers into Pluronic gel matrixes is also a well explored approach to improve the therapeutic properties of different drugs, as recently reviewed by Jaquilin et al. [119].

**Optimization of drug-loading process**. Drug-loaded micelles can be prepared following different procedures that include direct dissolution, dialysis, solvent evaporation or solid dispersion [120]. The easiest and safest method is direct dissolution, as no organic solvents are required. However, the drug loading capacity of these micelles is relatively low. To increase the drug loading, a dialysis or evaporation method can be used, dissolving the drug and the polymer in an organic solvent, and then removing it [121]. Sotoudegan et al. prepared nimodipine-loaded micelles with Pluronics P85, F127 and F68 using two different approaches, direct dissolution and thin-film hydration, in two different organic solvents—methanol and acetonitrile [122]. The drug loading capacity was slightly higher in micelles prepared by direct dissolution, while thin-layer hydration led to more unstable micelles as they tended to form bigger aggregates. In contrast with methanol, acetonitrile caused the formation of bigger micelles, but decreased the amount of the encapsulated drug. Stammet et al. demonstrated that the drug loading method can generate different types of aggregates in a solution, which may influence the drug loading efficiency [123]. For example, solvent casting of resveratrol on various Pluronics was more efficient than loading through an equilibrium method. In addition, slight modifications in the traditional methods may optimize the preparation of micelles. Wang et al. described that the use of a central composite design method improved the production of chrysophanol-loaded Pluronic F127 micelles by dialysis [124]. On the other hand, it is interesting to note that the preparation sequency may affect the final self-assembled structure obtained, even when using the same composition, modifying the drug releasing profile [113]. 

**Covalent attachment of cargo to the polymer**. The covalent attachment of drugs to the polymer used to form the micelles may lead to an increased drug loading [121], but also an improved targeting to diseased tissue. Pellosi et al. developed core–shell NPs based on the self-assembly of rhodamine-conjugated P85, as a labelled polymer, and biotynilated-F127, as a targeting unit [125]. After loading with the drug temozolomide and the photosensitizer verteporfin, they confirmed their promising action in the treatment of glioblastoma multiforme. Wang et al. generated mixed micelles based on dexamethasone-conjugated P123 with redox and pH-responsive properties as a new strategy to target the cell nucleus [126].

## 6. Conclusions

Pluronic-based nanotechnology is a fast-flourishing field with applications in different areas, mainly pharmaceutics, cosmetics and veterinary. Due to their structural simplicity and versatility, Pluronic direct and reverse micelles stand out as interesting drug delivery systems that increase the drug solubility, the blood circulation time and the cellular uptake, but with low cytotoxicity. Nevertheless, micellization is not a straightforward process and understanding the factors governing this is fundamental to obtaining a product that reaches clinical applications. Herein, we highlighted six factors: (1) The Pluronic length, concentration and PEO:PPO ratio; (2) the use of normal or reverse Pluronics, which exhibit a different ability to form regular micelles but whose combination appears as a promising approach to increasing the micelles’ stability and drug loading; (3) the solvents, which can favor or disfavor the micellization process depending on the ability to solvate the different blocks in the Pluronic; (4) the temperature, which clearly affects the CMC and the hydrodynamic diameter of Pluronic micelles; (5) the pH and ionic strength, easily tuned by the addition of salts or hydrotropes, which influence the micelle formation, size and shape; and finally, (6) the nature of the loaded cargo, where highly lipophilic drugs increase the micelle’s size and ionizable cargo can generate pH-responsive nanocarriers that modulate the drug release.

Despite the complexity of the micellization of Pluronics, accurate information can be obtained through a myriad of techniques, as summarized in Table 4: scattering techniques, spectroscopic methods, calorimetric approaches, microscopic techniques, rheological strategies, etc. The most relevant information is obtained by a thorough and complementary use of these techniques, which provides light not only on the micellar structure but also on their ability to load and release relevant cargo.

Regarding the transfer from the bench to the clinic, Pluronic micelles currently face two main drawbacks: stability issues under physiological environments, which leads to a premature release of the drug, and relatively low drug encapsulation, limiting their use to drugs with high therapeutic potency. Furthermore, the broad variability of Pluronic polymers require a detailed study of their toxicity. As herein reported, there is intense research on Pluronics technology, which provides innovative solutions to overcome these drawbacks. A smart design of the polymer precursor is crucial as well as considering all the previous factors influencing the micellization (solvents, additives, temperature, etc.). Furthermore, relevant strategies that can substantially improve the stability and cargo loading of these nanosized drug delivery systems are currently in the spotlight. For example, the formation of mixed micelles based on different Pluronics or other surfactants, broadly explored in current literature; the assembly of individual micelles into nanogels or the dispersion of these micelles in Pluronic hydrogels to increase the residence time in a specific tissue; or the crosslinking of the micellar shell or core, and subsequent release upon exposure to a specific stimuli. In this context, Pluronic reverse micelles also arise as an underexplored tool for the delivery of sensitive cargo. 

Overall, Pluronic micelles present a bright future as drug delivery agents. A smart design and thorough characterization of the nanosized vehicles, together with the innovative strategies arising to overcome current drawbacks, will undoubtedly improve the transfer to clinical applications in the upcoming years. 

## Figures and Tables

**Table 2:** Summary of effects of different solvents on Pluronic micellization. Positive: favors the formation of micelles. Negative: disfavors the formation of micelles. All mixtures employ water as main solvent.

	Solvent/s	Effect on Micellization
Binary mixtures	Short-chain alcohols (ethanol) [28,33]	Negative
	Medium-chain alcohols (butanol, glycerol) [28]	Positive
	Oils (*p*-xylene) [29,30]	Positive (different assemblies)
	Glucose [33]	Positive
	Propylene carbonate [33]	Positive
	Triacetin [33]	Positive
	DMSO [35]	Positive
	Ionic liquids (ethylammonium nitrate) [12]	Positive
Ternary mixtures	Ethanol/glycerol [33]	No effect
	Ethanol/propylene carbonate [33]	No effect
	Ethanol/turpentine oil [34]	Positive (different assemblies)

## Data Availability

Not applicable.
